# Dydrogesterone in the treatment of endometriosis: evidence mapping and meta-analysis

**DOI:** 10.1007/s00404-020-05900-z

**Published:** 2021-01-04

**Authors:** Chao Peng, Yan Huang, Yingfang Zhou

**Affiliations:** Department of Obstetrics and Gynecology, Peking University First Hospital, No. 8 Xishiku Street, Xicheng District, Beijing, China

**Keywords:** Dydrogesterone, Endometriosis, Evidence mapping, Gestrinone, GnRH agonist

## Abstract

**Purpose:**

Endometriosis is a common, chronic gynecological disease that affects women’s fertility potential. Dydrogesterone is an effective and safe drug that is under-utilized due to limited clinical research. The purpose of this evidence mapping is to identify, describe, and analyze the current available evidence regarding dydrogesterone for the treatment of endometriosis.

**Materials and methods:**

We performed a search in electronic databases: Medline, The Cochrane Library, EMBASE, PubMed, CNKI, Wanfang, VIP, and CBM. We also hand-searched google for relevant studies. Our primary outcomes included changes in pain relief including pelvic pain, dysmenorrhea, and dyspareunia. Secondary outcomes included pregnancy rate, frequency of analgesic use, and other reported outcomes according to specific settings in the studies.

**Results:**

Of 377 references screened, 19 studies were included in the data synthesis involving 1709 female participants. Nearly three-quarters were either randomized control trials or clinical control trials. Compared with gestrinone, dydrogesterone relieved dysmenorrhea, increased the pregnancy rate, and reduced the risk of certain adverse events. Compared with GnRH-a, dydrogesterone also lowered the risk of endometriosis recurrence and elevated transaminase levels. Whether there was any difference in efficacy between dydrogesterone and leuprolide acetate, letrozole or traditional Chinese medicine remains unclear due to insufficient data.

**Conclusions:**

The amount and quality of evidence evaluating the effects of dydrogesterone for the treatment of endometriosis is generally very low. Limited evidence suggests that dydrogesterone may have some advantages over gestrinone, GnRH agonists, and other therapeutic interventions in treating endometriosis. However, this conclusion should be interpreted with caution.

## Introduction

Endometriosis, defined as the presence of endometrial-like tissue outside the uterus, is a complex and chronic gynecological disease that affects women’s fertility potential [1]. The prevalence of endometriosis has been estimated to be between 2 and 10% for women of reproductive age, and between 25 and 50% for women with infertility [2, 3]. Although patients with endometriosis may be asymptomatic, most patients usually present with one or more associated symptoms, including dysmenorrhea, chronic pelvic pain, deep dyspareunia, cyclical intestinal complaints, fatigue/weariness, and infertility [1]. Endometriosis-associated symptoms progressively impair the ability of women to carry out certain daily activities and result in worsening health status and overall well-being [4]. In addition, 2–4% of women who are sexually active may have sexual dysfunction caused by this disease [5]. Endometriosis is also associated with considerable direct and indirect costs that is comparable to those resulting from major global chronic diseases such as diabetes. Finally, endometriosis-related symptoms substantially interfere with the employment of affected women, often resulting in several missed work days [4].

The etiology of endometriosis remains obscure. The development of endometriosis is a complex process with a large number of interconnected factors that may be both inherited and acquired [6]. Accumulating evidence suggests that immune cells, adhesion molecules, extracellular matrix metalloproteinase and pro-inflammatory cytokines activate or alter the peritoneal microenvironment, creating the conditions for differentiation, adhesion, proliferation, and survival of ectopic endometrial cells. New theories about the pathogenesis of endometriosis suggest it may originate from Müllerian or non-Müllerian stem cells, including those from the endometrial basal layer, Müllerian remnants, bone marrow, or the peritoneum. The innate ability of endometrial stem cells to regenerate cyclically also seems to play a key role. There is also evidence to support the hypothesis that ectopic Müllerian remnants of the endometrium, endocervix, and endosalpinx are ‘leaked’ from the genital ridge during organogenesis [7]. The dysregulation of hormonal pathways, as evidenced by increased estradiol production and progesterone resistance observed in women with endometriosis, has been a widely accepted theory about the pathogenesis of endometriosis [8].

It is now accepted that inflammation clearly plays a central role in the development and progression of endometriosis and is characterized by the overproduction of an array of inflammatory mediators such as prostaglandins, metalloproteinases, cytokines, and chemokines. The growth and adhesion of endometrial cells in the peritoneal cavity due to reactive oxygen species (ROS) and free radicals is thought to lead to disease onset, with its ensuing symptoms of pain and resultant infertility [9].

Symptomatic endometriosis remains the prime indication for treatment. Ideally, treatment should provide pain relief and allow pregnancy to occur safely while undergoing treatment. The current treatments for endometriosis include surgery (ablation using either laser or electrosurgery if laparoscopy is performed), pharmacological therapy, or a combination of both [10]. Symptomatic patients always receive pharmacological therapy, which can include: (i) analgesics for women with endometriosis-related pain, discuss the benefits and risks of analgesics, consider a short trial (for example, 3 months) paracetamol or a non-steroidal anti-inflammatory drug (NSAID) may provide adequate pain relief; (ii) hormonal treatments such as hormonal contraceptives, progestagens (e.g., progesterone), anti-progestagens (e.g., gestrinone), or gonadotropin-releasing hormone (GnRH) agonists (e.g., leuprolide) as it reduces endometriosis-associated pain [10]; (iii) alternative treatments: most recently, aromatase inhibitors (e.g., letrozole), traditional Chinese medicine, and acupuncture are considered potential therapies for endometriosis [11, 12]. The choice of drug therapy is essential and should offer relief from symptoms without inhibiting ovulation, causing amenorrhea or other adverse effects.

Dydrogesterone (6-dehydro-retroprogesterone) is a retroprogesterone derived from progesterone that is similar in structure and pharmacology to endogenous progesterone. It acts as a selective progesterone receptor agonist and has better oral bioavailability compared with oral micronized progesterone [13]. Dydrogesterone has been on the market since the 1960s and is used as postmenopausal hormone-replacement as well as for treatment of menstrual disorders and endometriosis [14]. Dydrogesterone has been shown to relieve symptoms of endometriosis, regress lesions, and improve pregnancy rates in patients with infertility [15].

The proposed mechanism underlying the pharmacological action of progestogens involves the initial decidualization of endometrial tissue and eventual atrophy. Dydrogesterone causes atrophy of ectopic endometrium without suppressing the normal endometrium and simultaneously inhibits the development of new endometriotic lesions [16]. Furthermore, it does not inhibit ovulation and regular menstruation and does not induce weight gain and edema [15]. However, one study showed that a 2 mg/day oral dosage of dienogest was more effective than a 10 mg twice daily oral dosage of dydrogesterone for relieving endometriosis-associated pelvic pain, with a comparable safety profile [17].

Early clinical studies evaluating dydrogesterone efficacy and safety have been limited by small sample sizes and a lack of direct comparisons with control groups. Therefore, the aim of our study is to search and analyze available evidence surrounding the efficacy and safety of dydrogesterone in the treatment of endometriosis. To consolidate knowledge, avoid scientific redundancies, and identify research gaps, we provide a mapping of the empirical literature on the effects of dydrogesterone in the treatment of endometriosis.

## Materials and methods

Evidence mapping of empirical literature on the effects of dydrogesterone in the treatment of endometriosis was performed.

### Data source

We searched in the following electronic databases: Medline, The Cochrane Library, EMBASE, PubMed, CNKI, Wanfang, VIP, and CBM from inception to September 19, 2019. There was no limitation on publication status, publication dates, or language. The search strategy used in each database is presented in Appendix 1. We also hand-searched google for relevant studies.

### Study design

All randomized control trials (RCTs), clinical control trials (CCTs), and observational studies were included in the evidence map.

### Participants

We relied on the diagnosis of endometriosis as presented in the included studies.

### Interventions and comparisons

Any studies that evaluated dydrogesterone alone were included in this evidence map without limitations regarding treatment dosage, frequency, and duration. There were no limitations on the number or types of the comparison. Furthermore, all single-arm studies without comparator were also included.

### Outcomes

Primary outcomes included changes in pain relief including pelvic pain, dysmenorrhea, and dyspareunia. Secondary outcomes included the pregnancy rate, frequency of analgesic use, and other reported outcomes according to specific settings in the studies.

### Exclusion criteria

- Studies that were other than RCTs, CCTs, or observational studies;
- Patients who were not diagnosed with endometriosis;
- Studies that did not include dydrogesterone therapy;
- Interventions that combined dydrogesterone with other therapies;
- Language that was other than Chinese or English;
- Studies with only an abstract and no full text.

### Study selection process

**Fig. 1:**
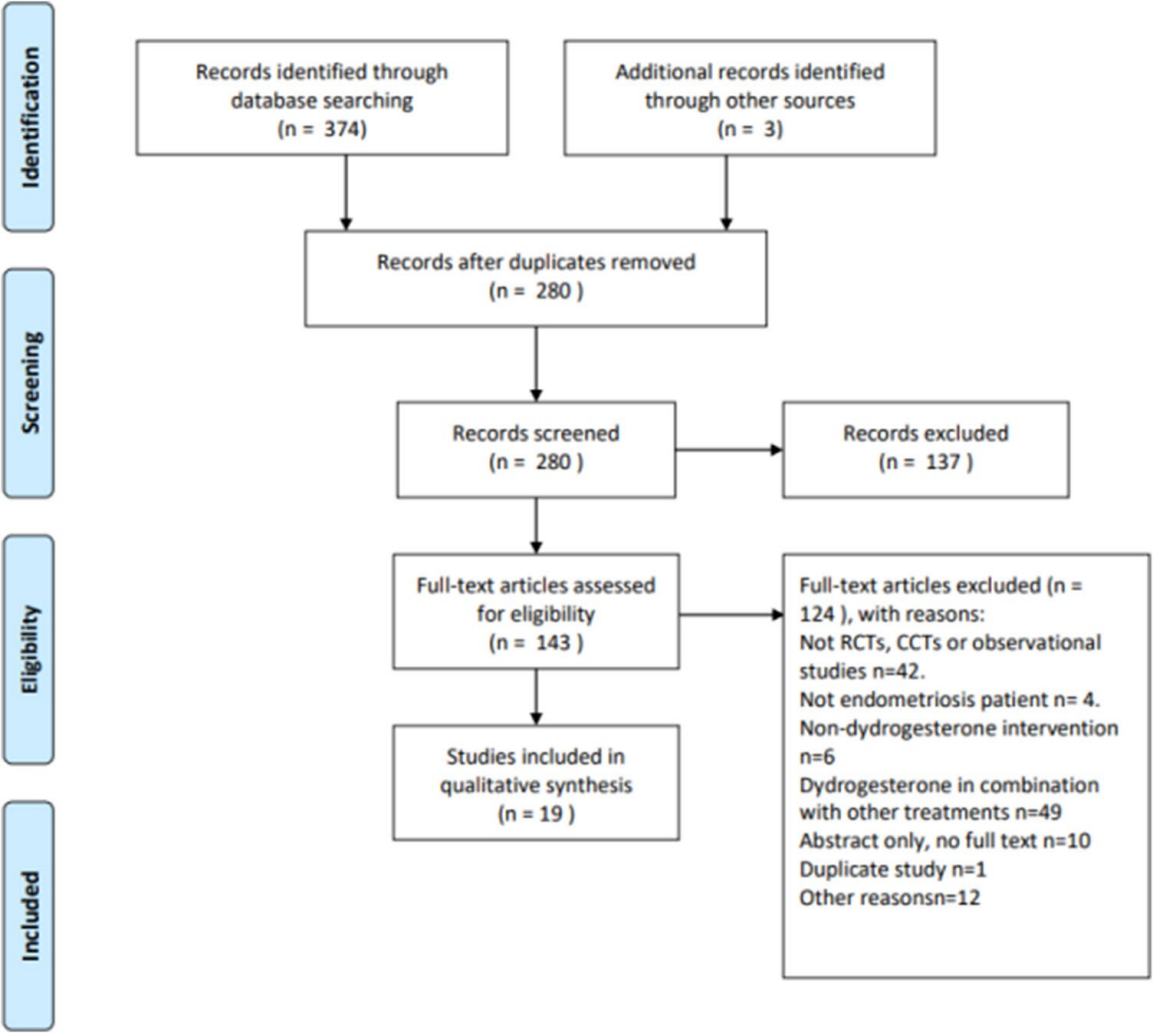
PRISMA flow diagram

Two reviewers (CP, YH) screened the search results. All potentially relevant citations were requested and inspected in detail via the full-text version. Disagreements were resolved by discussion with assistance from a third party (YFZ) if necessary. A PRISMA flow diagram was constructed to show the full study-selection process (Fig. 1).

### Data extraction

Data from each study were extracted independently by two separate reviewers. A standardized data extraction form was designed and tested using a pilot data extraction exercise. Any disagreements were resolved by discussion with assistance from a third party if necessary. Where more information relating to a potentially includable study was lacking, we contacted study authors and requested further information. We extracted all relevant characteristics of included studies including:

- General study characteristics (first authors, publication years, study location, center, and sample size);
- Population characteristics including diagnosis, age, settings, inclusion and exclusion criteria;
- Intervention characteristics including administration of interventions and treatment duration;
- Outcome characteristics such as outcome category, definition of the outcome, and the time point of the measurement;
- Key findings of each study.

### Data synthesis and analysis

We used Revman 5.3 to conduct the meta-analysis. Before performing the meta-analysis, studies were judged homogeneous in terms of the characteristics of the study population, intervention used, outcomes, study design, and statistical metric. We used a random-effects model to pool the data. Statistical heterogeneity between the summary data was evaluated using the *I*^2^ statistic (≤ 25% represents insignificant heterogeneity, 26–50% represents low heterogeneity, 51–75% represents moderate heterogeneity, and ≥ 75% represents high heterogeneity). Where moderate/high statistical heterogeneities (*I*^2^ > 50%) were found, we explored the source of heterogeneity and tried to identify its cause. A subgroup analysis was performed if the causes of heterogeneity were identified. When the source or cause that induced heterogeneity could not be identified, we synthesized data using a random-effects model, and our confidence on the study findings was downgraded. We assessed publication bias by examining funnel plots when the number of trials reporting the outcomes was ten or more [18].

### Risk of bias assessment

The risk of bias of included studies was assessed using the Cochrane risk of bias tool for interventional studies. The domains of risk of bias assessed included randomization, allocation concealment, blinding, study attrition, selective reporting, and other bias. We also provided an overall assessment of each study. We rated a trial low risk of bias when all risk of bias domains were assessed as low risk, moderate risk of bias when at least one domain was assessed as moderate with no high risk assessments, and high risk of bias when any domain was assessed as high risk.

The cohort study included was assessed using Newcastle–Ottawa Scale (NOS) for evaluating selection, comparability, and outcome.

Study assessments were performed by CP and YH, and disagreements were resolved by discussion with assistance from a third party YFZ if necessary.

## Results

### Mapping of included evidence

The trial search identified 377 references, and 280 references remained after removing duplicates. A total of 137 citations were excluded after screening of the title and abstract. Subsequently, 124 articles were excluded following a full-text review, leaving 19 articles eligible for qualitative synthesis [12, 16, 19–35]. The study screening process and reasons for exclusion at the full-text screening stage are presented in Fig. 1.

### Summary of publication years

**Fig. 2:**
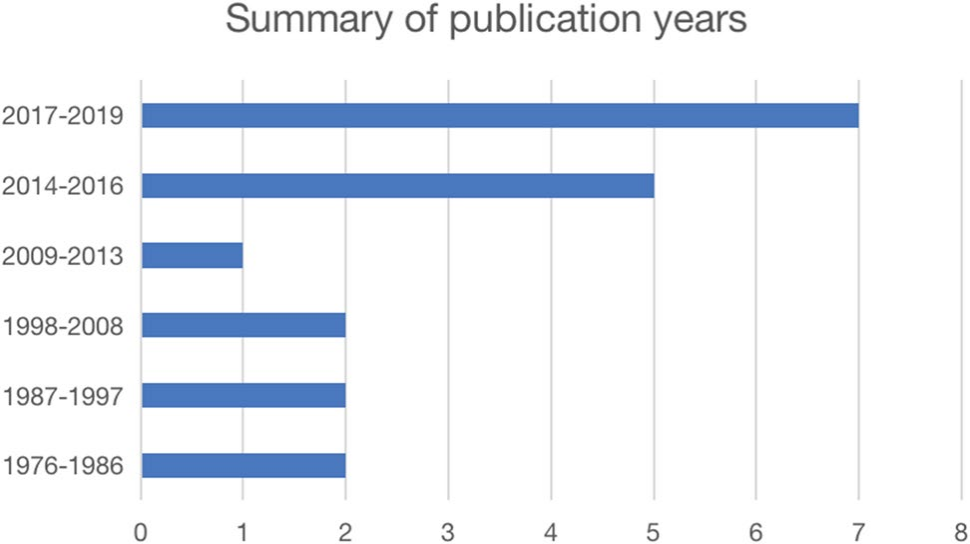
Number of included studies by year of publication

The earliest included study was published in 1976. More than half of the included evidence were published from 2014 to 2019 (Fig. 2).

### Summary of studies

In all, 19 studies [12, 16, 19–35] were included. Nine RCTs were identified that compared dydrogesterone with a different dosage of dydrogesterone or placebo (1 study [20] with 62 participants), gestrinone (7 studies [22, 24, 25, 27, 28, 30, 32] with 693 participants), [20] or traditional Chinese medicine and acupuncture therapy (1 study ^[12]^ with 64 participants). Four CCTs were identified that compared dydrogesterone with letrozole (1 study [26] with 90 participants), gestrinone (1 study [29] with 120 participants), GnRH-a leuprolide (1 study [31] with 80 participants), or coagulation of endometriotic foci, danazol, norcolut and depo-medroxyprogesterone acetate (1 study with 300 participants) [35]. One cohort study [33] was identified that compared dydrogesterone with no treatment after surgery (with 69 participants). The remaining 5 studies [16, 19, 21, 23, 34] (with 231 participants) were single-arm studies that investigated dydrogesterone without comparisons.

Included studies originated from various regions. A majority of studies (63%) were conducted in China, followed by Australia (5.3%), United Kingdom (10.5%), India (5.3%), Russia (5.3%), Belgium (5.3%), and Uzbekistan (5.3%). The number of patients in each study ranged from 18 to 130, for a total of 1709 patients. Most of the studies were conducted in a single center (73.6%), and most did not provide funding information (89.5%) (Table 1).

**Table 1:** Mapping of general study characteristics (total *N* of included records = 19)

Item	Variables	*N* of studies	% of studies
Study design	RCT	9	47.3
	CCT	4	21.1
	Cohort	1	5.3
	Single-arm study	5	26.3
Region	Australia	1	5.3
	United Kingdom	2	10.5
	Russia	1	5.3
	India	1	5.3
	Belgium	1	5.3
	China	12	63
	Uzbekistan	1	5.3
Sample size	Range	14–300	–
	≥ 100	5	26.3
Center	Single	14	73.6
	Multi	4	21.1
	NR	1	5.3
Funding	Industry	0	0
	Non-industry	2	10.5
	NR	17	89.5

### Summary of population characteristics

Across the 19 included studies, the age of participants ranged from 18 to 51 years (mean age from 28.8 to 35.2 years). Twelve studies [12, 16, 19–21, 24–26, 31, 33–35] used pathological examination and surgery—culdoscopy, laparoscopy, and laparotomy—to diagnose endometriosis. Three studies [20, 21, 33] used a diagnosis of endometriosis in line with the AFS classification, and one study [31] used a diagnosis in line with the Guide for Diagnosis and Treatment of Endometriosis. One study [29] used a mix of color Doppler ultrasound and surgery consistent with the criteria for diagnosing endometriosis presented in the journal Obstetrics and Gynecology published by the People’s Health Publishing House. One study [32] used a mix of electrocoagulation, pathological examination, and surgery to diagnose endometriosis, while another study [12] used solely the diagnostic criteria put forth in the Clinical Diagnosis and Treatment Guide—Obstetrics and Gynecology Volume (People’s Medical Publishing House, 2011). The remaining five studies [22, 23, 27, 28, 30] did not provide information on the diagnostic criteria used.

### Summary of intervention in single-arm studies

Five included single-arm studies [16, 19, 21, 23, 34] investigated various dosages of dydrogesterone and different treatment durations. Two studies [16, 21] prescribed 10–20-mg dydrogesterone daily to patients according to the severity of endometriosis (40%). The other three studies [19, 23, 34] prescribed 20–30-mg or 20–60-mg dydrogesterone daily (60%). Four studies [16, 19, 21, 34] reported various treatment durations, and one study [23] did not report treatment duration (Table 2).

**Table 2:** Mapping of single-arm studies (total N of studies = 5)

Study ID	Country	Sample size	Mean age	Intervention	Outcomes reported	Key findings
Cornillie [19]	Belgium	18	NR	Dydrogesterone 20–60 mg/day for 2–5 months	Endometriosis improvement—change in AFS score (5 months), efficacy (5 months), pregnancy (12 months), clinical response—microscopic findings in left ovary (5 months)	• Neither in pre-treatment nor in post-treatment biopsies was there a clear correlation between the cytologic differentiation of ectopic and ectopic endometrium • Secretory changes within ectopic foci were seen in only some of the biopsies from patients with a secretory endometrium both before and after treatment
Ji [23]	China	60	30.6	Dydrogesterone 20–30 mg/day on Day 5–25 of each cycle	Efficacy (after treatment), endometriosis improvement	• Of 60 patients with endometriosis, all but 4 had improvement after treatment (90%) compared with the condition before treatment • The discomfort response in the body of patients gradually decreased, recovered to health, and the quality of life was significantly improved after treatment • The physical signs, condition performance and serum hormone levels of the patient were significantly better than that of the patients before treatment
Johnston [16]	Australia	49	NR	Dydrogesterone 10 mg/day for 9 months	Efficacy (9 months), relief on symptoms (3–4 months), recurrence (15 months), pregnancy (9 months), appearance of uncharted lesions (9 months), impediment to fertility (9 months), adverse events (9 months)	• Dydrogesterone, by a mechanism which is not known, causes atrophy and disappearance of areas of ectopic endometrium at a dose which does not give rise to atrophy or suppression of endometrium within the endometrial cavity • Of 49 patients with endometriosis, all but five had no subjective symptoms after 9 months treatment
Trivedi [21]	India	98	30.85	Dydrogesterone 10–20 mg/day on Day 5–25 of each cycle for 3–6 months	Pain relief—change in visual analogue scale scores (6 months), relief on symptoms (6 months), adverse events efficacy (6 months), pregnancy (6 months), score for amount of bleeding (6 months), duration of bleeding (6 months)	• Dydrogesterone is an effective and safe post-laparoscopic treatment for endometriosis • Dydrogesterone for the treatment of post-laparoscopic endometriosis, statistically significant reductions in the symptoms pelvic pain, dysmenorrhea and dyspareunia were seen after the first treatment cycle • The amount and duration of menstrual bleeding was also significantly reduced, and from the end of the third month onwards, bleeding was considered normal in the majority of patients • Improvement of endometriosis was observed in 71% of patients and cure in 21% • No adverse events were reported
Walker (1983)	United Kingdom	14	NR	Dydrogesterone 30 mg/day on Day 5–25 of each cycle for 6 months	Relief on symptoms (6 months), efficacy (6 months)	• Six of ten patients showed a significant improvement in symptoms • Dydrogesterone given cyclically on days 5–25 in a dose of 10 mg three times a day is effective in the treatment of pelvic pain associated with endometriosis

### Summary of outcomes and findings in single-arm studies

The outcomes and measurements or definitions of the assessments reported in the five single-arm studies [16, 19, 21, 23, 34] are presented in Table 2.

Overall, four studies (80%) [19, 21, 23, 34] reported changes in pain relief. Three studies (60%) measured the pregnancy rate [16, 19, 21]. All five studies (100%) [16, 19, 21, 23, 34] measured clinical response. One study (20%) [16] measured the recurrence rate [6]. Two studies (40%) [16, 34] assessed improvement in endometriosis. Another two studies (40%) [16, 23] reported adverse events [6]. One study (20%) [16] measured the appearance of uncharted lesions and the impediment to fertility [6], and one study (20%) [34] measured the duration of the menstrual cycle. The key findings of the five single-arm studies are summarized in Table 2.

### Summary of intervention and comparators in RCTs and CCTs and the cohort study

Nine RCTs [12, 20, 22, 24, 25, 27, 28, 30, 32], four CCTs [26, 29, 31, 34] and one cohort study [33] compared the effect of various dosages of dydrogesterone versus non-dydrogesterone therapy (Table 3). One study [20] compared a low (40 mg/day)and high dosage (60 mg/day) of dydrogesterone versus placebo. The remaining 13 studies [12, 22, 24–34] compared dydrogesterone (10–20 mg/day) with non-dydrogesterone therapies, namely gestrinone (*n* = 8), letrozole (*n* = 1), GnRH-a leuprolide acetate (*n* = 1), traditional Chinese medicine (*n* = 1), no treatment (*n* = 1), coagulation of endometriotic foci, danazol, norcolut and depo-medroxyprogesterone (*n* = 1). Of these 13 studies, 8 [22, 24, 25, 27–30, 32] evaluated gestrinone, and participants were administered 2.5 mg twice a week from day 1 of menstruation after surgery for a duration of 3 months (*n* = 4) [24, 29, 30, 32], 6 months (*n* = 1) [28], or 3–6 months (*n* = 3) [22, 25, 27].

**Table 3:** Mapping of study characteristics, interventions and comparators in RCTs, CCTs and cohort study (total *N* of studies = 14)

Study ID	Study design	Country	Sample size	Mean age	Intervention	Study risk of bias	Outcomes reported	Time point measured
Feng [22]	RCT	China	116	Dydrogesterone: 35.1; gestrinone: 34.0	Dydrogesterone 10–20 mg/day vs gestrinone 5 mg/week	Moderate	Pain relief—change in VAS Adverse events Pregnancy	After treatment, 3–6 months NR NR
Li [25]	RCT	China	130	Dydrogesterone: 32.02; gestrinone: 32.76	Dydrogesterone 10–20 mg/day vs gestrinone 5 mg/week	Moderate	Pain relief—change in VAS Clinical response—serum CA-125 Recurrency Adverse events Pregnancy	3, 6, 12 months 3, 6, 12 months 12 months 12 months 12 months
Li [24]	RCT	China	75	Dydrogesterone: 31.5; gestrinone: 32.0	Dydrogesterone 20 mg/day vs gestrinone 5 mg/week	High	Efficacy Pregnancy Pain relief—change in VAS Adverse events	After treatment, 3, 12 months 12 months 3, 6, 12 months After treatment, 3, 12 months
Liu [28]	RCT	China	80	Dydrogesterone: 31.47; gestrinone: 31.83	Dydrogesterone 10–20 mg/day vs gestrinone 5 mg/week	Moderate	Recurrency Pregnancy Adverse events Pain relief—change in VAS Clinical response—hormone levels	12 months 12 months 12 months 12 months 12 months
Liu [27]	RCT	China	92	Gestrinone: 30.8; dydrogesterone: 30.1	Dydrogesterone 10–20 mg/day vs gestrinone 5 mg/week	Moderate	Pain relief—change in VAS Clinical response—serum CA-125 Adverse events Recurrency Pregnancy	3, 6 months 3, 6 months 3–6 months 12 months 12 months
Liu [12]	RCT	China	64	29.5	Dydrogesterone 20 mg/day vs traditional Chinese medicine and Chinese medicine acupoint	Moderate	Pain relief—change in VAS Efficacy Pregnancy Adverse events Miscarriage Allergic reaction or liver function damage	6 months 6 months 12 months NR 12 months NR
Overton [20]	RCT	United Kingdom	62	30	Dydrogesterone 40 mg/day vs 60 mg/day vs placebo	Moderate	Endometriosis improvement—change in AFS score Pregnancy Pain relief—change in VAS	3 months 12 months 12 months
Wang [30]	RCT	China	80	Gestrinone: 34.22; dydrogesterone: 35.36	Dydrogesterone 10 mg/2 days vs gestrinone 5 mg/week	Moderate	Clinical response—serum CA-125	After treatment, 3 months
Zhang [32]	RCT	China	120	30.5	Dydrogesterone 20 mg/day vs gestrinone 5 mg/week	Moderate	Efficacy Adverse events Recurrency Menstrual recovery time Pregnancy Pain relief Pelvic nodules Ovarian chocolate cysts	After treatment, 3 months After treatment, 3 months 24 months 24 months 12, 24 months NR NR NR
Li [26]	CCT	China	90	Letrozole: 35.1; duphaston: 35.2	Dydrogesterone 20 mg/day vs letrozole 2.5 mg/day	High	Efficacy Clinical response—serum IL-6, TNF-α, VEGF	NR After treatment, 6 months
Luo [29]	CCT	China	120	Dydrogesterone: 28.8; gestrinone: 29.4	Dydrogesterone 20 mg/day vs gestrinone 5 mg/week	High	Clinical response—serum CA-125 Clinical response—hormone levels Pain relief—change in VAS Adverse events	After treatment, 3 months After treatment, 3 months After treatment, 3 months NR
Makhmudova [35]	CCT	Uzbekistan	300	31	Dydrogesterone 10 mg/day vs coagulation of endometriotic foci vs danazol 800 mg/day vs norcolut 10 mg/day vs depo-medroxyprogesterone 50 mg/week	High	Pregnancy	12 months
Xie [31]	CCT	China	80	34	Dydrogesterone 20 mg/day vs GnRH-a leuprolide acetate 3.75 mg/28 days	High	Recurrency Adverse events Clinical response—hormone levels Clinical response—serum CA-125 CA-199	12 months 3 months After treatment, 3 months After treatment, 3 months
Orazov [33]	Cohort	Russia	69	NR	Dydrogesterone 10 mg/day vs no treatment	Low	Pregnancy	6, 12 months

*RCT* randomized controlled trial, *CCT* controlled clinical trial, *VAS* visual analogue scale, *NR* not reported, *vs* versus

### Summary of outcome categories

Outcomes reported in the 14 randomized, clinical controlled and cohort studies [12, 20, 22, 24–34] are presented in Table 3.

Overall, nine studies (64.2%) reported changes in pain relief. Ten studies (71.4%) measured pregnancy rates. Nine studies (64.2%) measured clinical response using various definitions. Five studies (35.7%) measured the recurrence rate. Nine studies (64.2%) measured adverse events. One study (7.1%) evaluated improvement in endometriosis. One study (7.1%) assessed menstrual function disorders. Only one study (7.1%) measured menstrual recovery time. One study (7.1%) assessed dyspareunia. One study (7.1%) measured the number of miscarriages. Two studies (14.2%) assessed allergic reactions or liver function damage, pelvic nodules, and the incidence of ovarian chocolate cysts.

### Summary of key findings in comparative studies

Overton et al.  [20]  compared dydrogesterone (40 mg/day), dydrogesterone (60 mg/day) with identical placebos and found that pain was significantly relieved after treatment with 60-mg dydrogesterone for 6 months. Furthermore, this improvement was still evident at the 12-month follow-up. No differences were identified in the change in pain score with 40 mg of dydrogesterone compared with placebo (OR 0.80, 95% CI 0.27–2.37). There was no significant improvement in objective efficacy (AFS scores) at 6 months with dydrogesterone (40 mg and 60 mg) compared with placebo (OR 0.53, 95% CI 0.14–1.94). Even though a higher number of pregnancies was observed in the dydrogesterone group than in the placebo group (10/43 versus 3/19 at 6 months, 18/37 versus 7/19 at 12 months), this difference was not statistically significant. However, because of the wide confidence intervals, the data should be interpreted with caution ^[36]^.

**Fig. 3:**
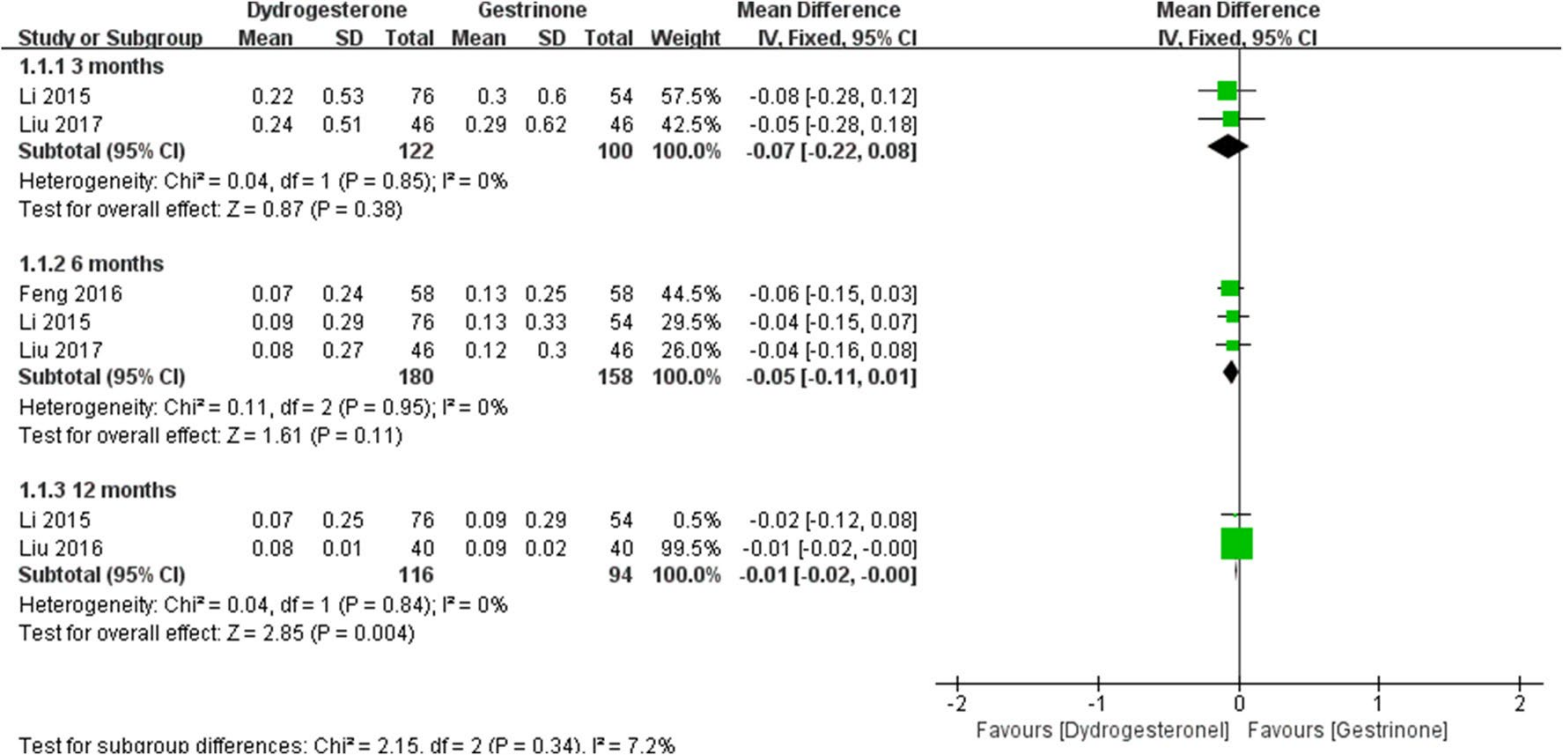
Meta-analysis of dysmenorrhea: dydrogesterone versus gestrinone

**Fig. 4:**
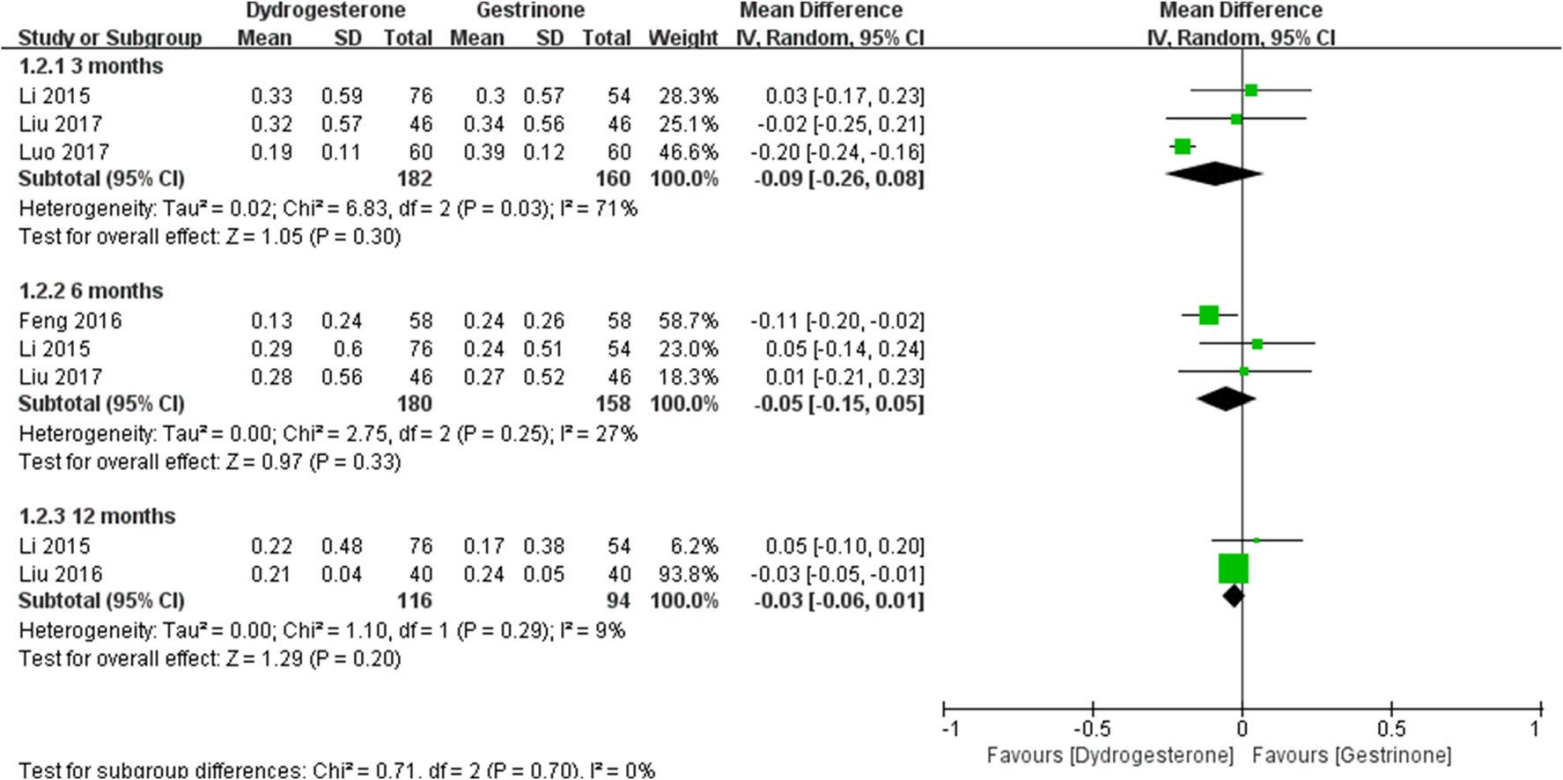
Meta-analysis of pelvic pain: dydrogesterone versus gestrinone

**Fig. 5:**
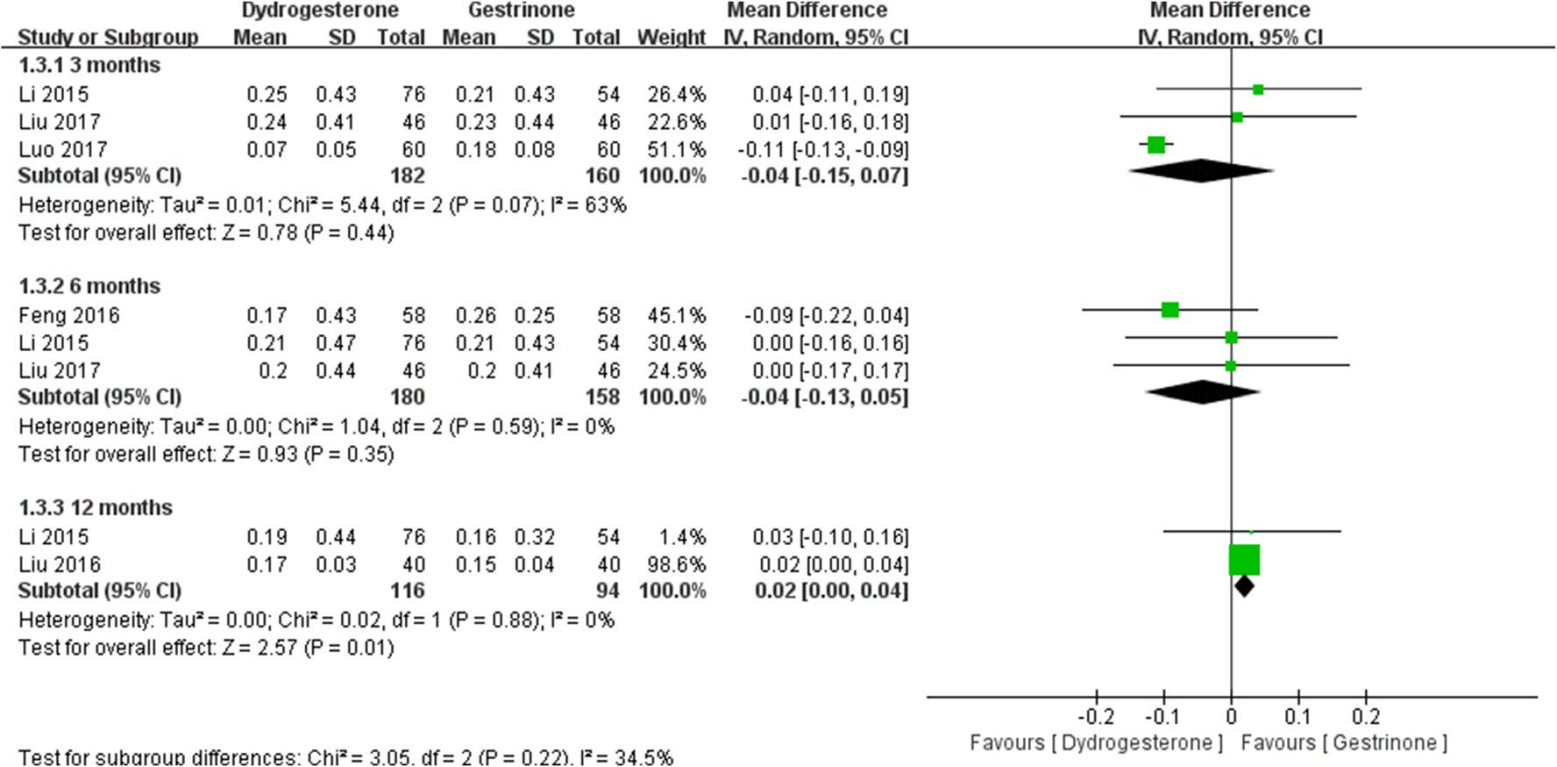
Meta-analysis of dyspareunia: dydrogesterone versus gestrinone

**Fig. 6:**
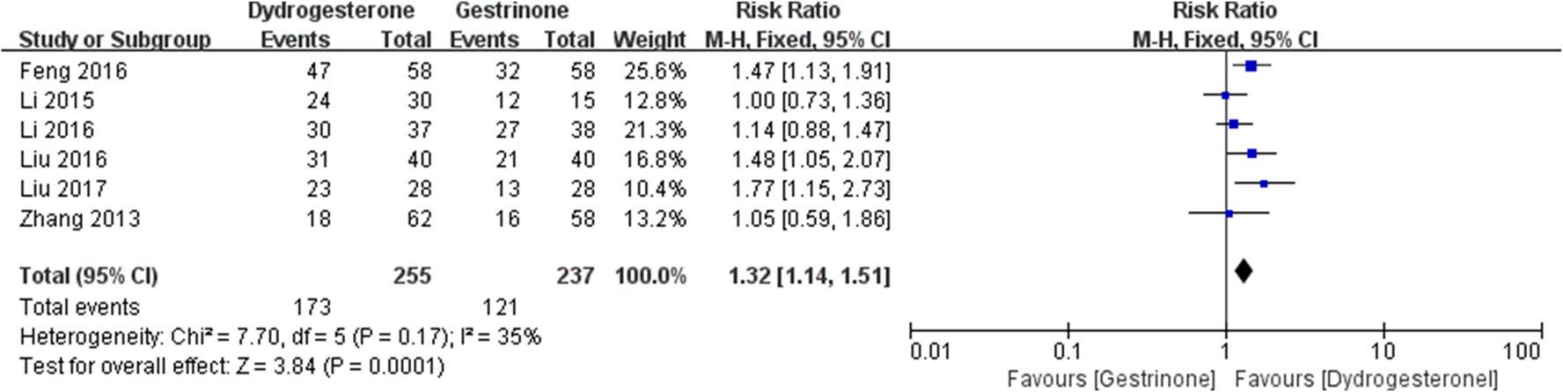
Meta-analysis of pregnancy rate: dydrogesterone versus gestrinone

**Fig. 7:**
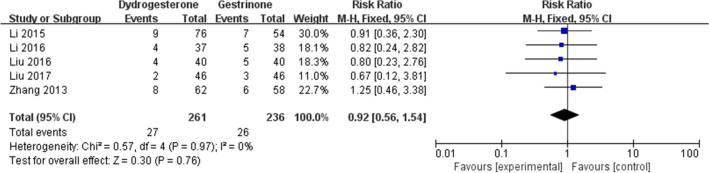
Meta-analysis of recurrence: dydrogesterone versus gestrinone

**Fig. 8:**
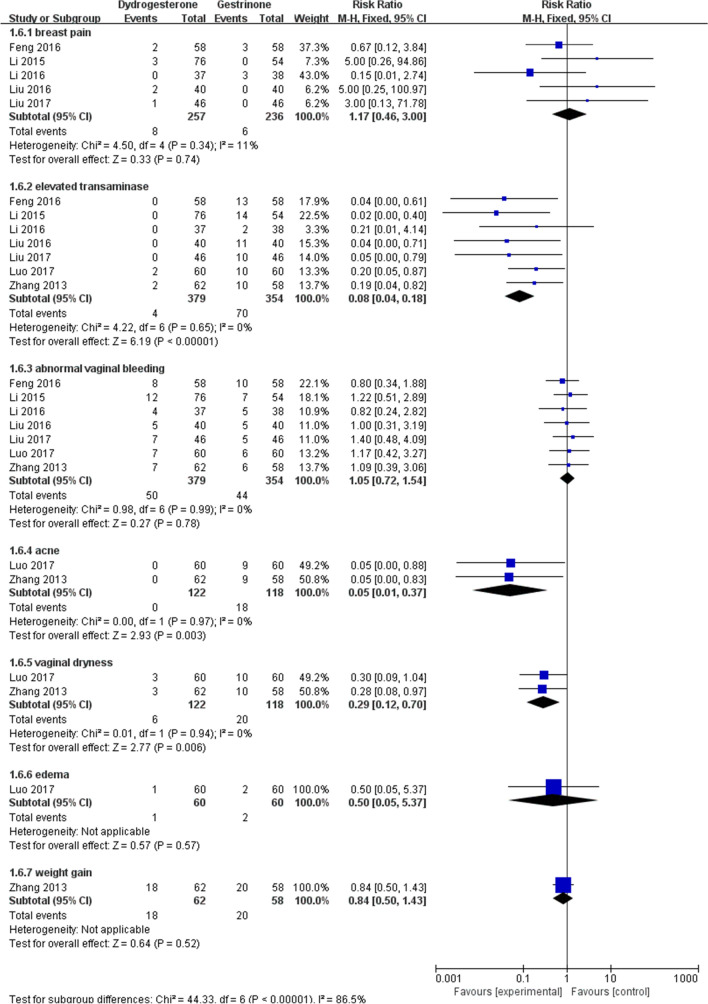
Meta-analysis of adverse events: dydrogesterone versus gestrinone

**Fig. 9:**
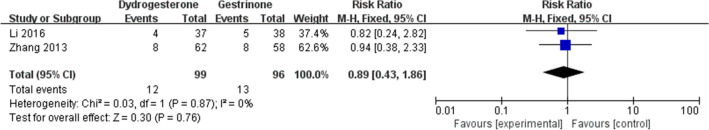
Meta-analysis of no clinical response: dydrogesterone versus gestrinone

Eight studies [22, 24, 27–30, 32] compared dydrogesterone versus gestrinone. Compared with the gestrinone group, patients treated with dydrogesterone had statistically lower VAS of dysmenorrhea after treatment for 12 months (Fig. 3). There was no significant difference between the groups in the occurrence of dysmenorrhea at 3 and 6 months (Fig. 3, or pelvic pain and dyspareunia 3, 6 and 12 months after treatment (Fig. 4, 5). Moderate heterogeneity (*I*^2^ = 71%, p = 0.03 and *I*^2^ = 63%, p = 0.07, respectively) was observed for pelvic pain and dyspareunia at 3 months, which was caused by the Luo 2017 study. This was probably due to the use of a smaller dose (10 mg versus 10–20 mg) and the shorter duration (3 month versus 3–6 months) of dydrogesterone use. After treatment, patients treated with dydrogesterone had much higher pregnancy rates than those receiving gestrinone (Fig. 6). No significant difference was found in the recurrence rate of endometriosis between the groups (Fig. 7). In addition, the rate of adverse events (elevated transaminase levels, vaginal dryness, and acne) was significantly lower in the patients treated with dydrogesterone than gestrinone (Fig. 8). Two studies [24, 32] in which clinical improvement of endometriosis were defined reported no difference between dydrogesterone and gestrinone treatment (Fig. 9).

One study compared dydrogesterone with GnRH-a leuprolide acetate [31], letrozole [26], traditional Chinese medicine [12], and no treatment [33]. The results are presented in Table 4. The study [33] comparing dydrogesterone with no treatment showed a favorable pregnancy rate in the dydrogesterone group after treatment, with a statistically significant improvement noted 6 months after treatment.

**Table 4:** Mapping of RCTs, CCTs and cohort study key findings (total *N* of studies = 14)

Outcomes	Definitions	Time point of measurement	Significant difference RR [95% CI]/*p* value	Favor of	*N* of participants	*N* (%) of studies
*Comparison 1: dydrogesterone (40 mg/day) versus dydrogesterone (60 mg/day)*						
Changes in pain relief	–	6 months	*p* = 0.044	Decrease of pain score in 60 mg dydrogesterone group	43	1 (7.1)
		12 months	*p* = 0.41	NS	43	1 (7.1)
Pregnancy rate	–	6 months	RR 0.77 [0.25, 2.34]	NS	43	1 (7.1)
		12 months	RR 1.05 [0.54, 2.04]	NS	43	1 (7.1)
Endometriosis improvement	AFS	3 months	OR 0.53 [0.14, 1.94]	NS	43	1 (7.1)
*Comparison 2: dydrogesterone versus gestrinone (results are presented in *Figs. 2, 3, 4, 5,6, 9, 10)						
*Comparison 3: dydrogesterone versus letrozole*						
Efficacy	Total = no improvements		RR 0.38 [0.11, 1.32]	NS	90	1 (7.1)
*Comparison 4: dydrogesterone versus GnRH-a leuprolide acetate*						
Recurrence	–	–	RR 0.13 [0.02, 0.95]	Lower risk of recurrence in dydrogesterone group	80	1 (7.1)
Adverse events	Breast pain		RR 1.00 [0.06, 15.44]	NS	80	1 (7.1)
	Elevated transaminase		RR 0.05 [0.00, 0.87]	Lower risk of elevated transaminase in dydrogesterone group	80	1 (7.1)
	Abnormal vaginal bleeding		RR 0.80 [0.23, 2.76]	NS	80	1 (7.1)
*Comparison 5: dydrogesterone versus traditional Chinese medicine and acupoint*						
Changes in pain relief—dysmenorrhea	VAS	6 months	0.1	NS	64	1 (7.1)
Pregnancy rate	–	12 months	RR 0.92 [0.54, 1.58]	NS	64	1 (7.1)
Miscarriage	–	12 months	RR 1.13 [0.07, 17.34]	NS	64	1 (7.1)
Efficacy	Total = no improvements		RR 1.26 [0.59, 2.68]	NS	64	1 (7.1)
*Comparison 6: dydrogesterone versus no treatment*						
Pregnancy rate	– –	6 months	RR 1.58 [0.75, 3.32]	NS	69	1 (7.1)
		12 months	RR 1.55 [1.00, 2.41]	In favor of dydrogesterone	69	1 (7.1)

*NS* no statistically significant difference

One study compared dydrogesterone (10 mg/day, n = 60) versus coagulation of endometriotic foci (during laparoscopy, n = 60), danazol (400 mg twice/day, n = 30), norcolut (10 mg/day, n = 60) and depo-medroxyprogesterone (50 mg/week, n = 60) ^[35]^. Danazol and dydrogesterone were the two most effective agents following surgical treatment in terms of the presence of pain, the restoration of a two-phase menstrual cycle, and the occurrence of pregnancy. However, no statistical inference was made in this study ^[35]^.

### Summary of validity of RCT and CCT studies

**Fig. 10:**
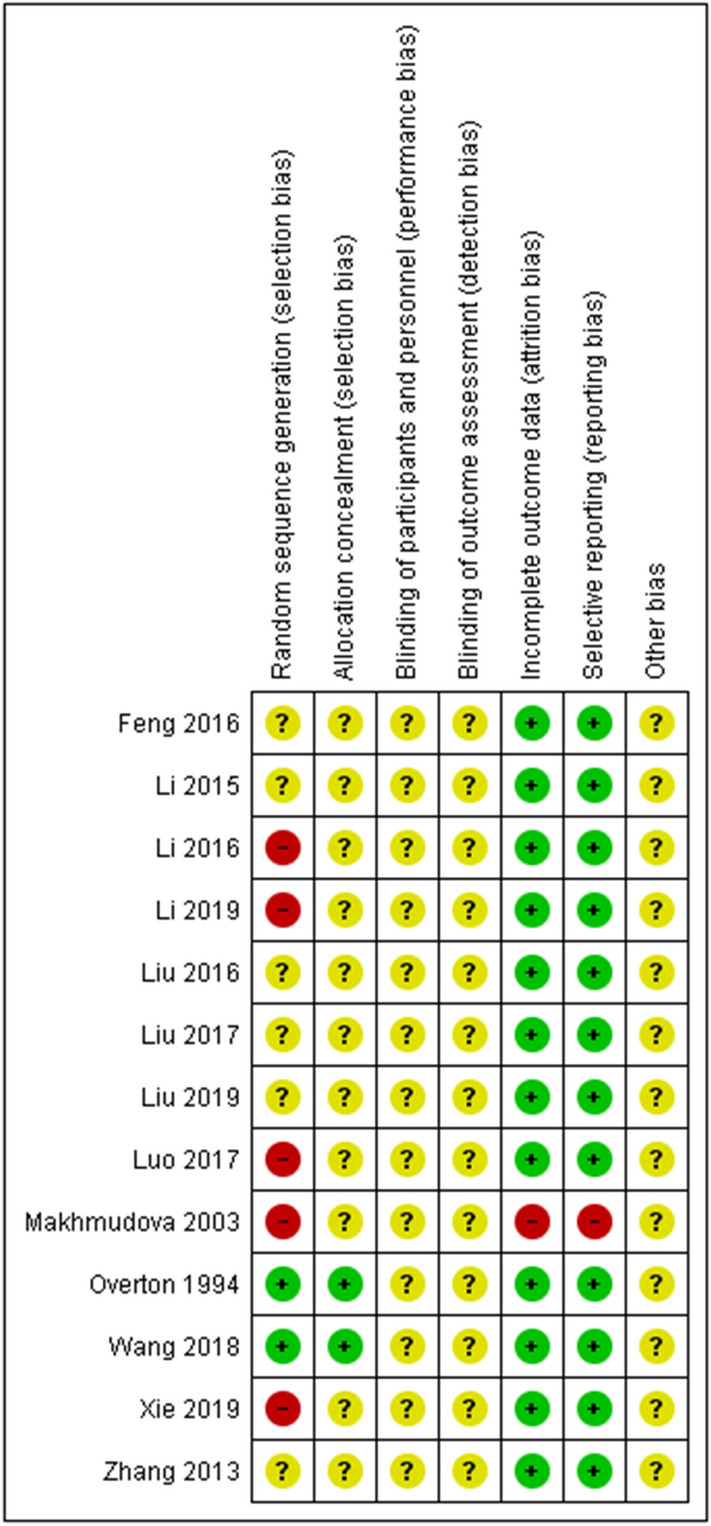
Mapping of RCT and CCT validity

The validity of studies was assessed for RCTs and CCTs using the Cochrane review standard—risk of bias assessment (Fig. 10).

#### Selection bias

In terms of random sequence generation and allocation concealment, only two studies were rated as low risk of selection bias by reporting a computer-generated randomization method. Four CCTs and one RCT were rated as high risk of selection bias. The remaining six studies were rated as unclear risk of bias because no randomization details were provided.

#### Performance bias

All 13 studies were rated as unclear risk of bias since no details about the blinding of participants and personnel were reported.

#### Detection bias

All 13 studies were rated as unclear risk of bias since no details about the blinding of the outcome assessment was reported.

#### Attrition bias

One study did not report withdrawals (through there may have been no withdrawals). The remaining 12 studies were rated as low risk of bias. Eleven studies of these 12 studies reported no withdraw during treatment and 1 of these 12 studies reported less than 10% drop-out rate.

#### Reporting bias

One study did not define the outcome clearly. The remaining 12 studies were rated as low risk of bias because all predefined outcomes were reported in the results.

#### Other bias

All 13 studies were rated as unclear risk of bias. Twelve studies did not report funding information, and 1 study reported non-industry funding.

### Validity of cohort study

Using the NOS assessment tool, a quality assessment of the included observational cohort study resulted in a score of 6 (2 points for selection, 1 point for comparability, and 2 points for outcome) which was consistent with a low risk of bias. Details of the assessment items in the domains for the included article are listed in Table 5.

**Table 5:** Newcastle–Ottawa quality assessment scale of cohort study

Study ID	Selection				Comparability	Outcome			Scores
	Representativeness of exposed cohort	Selection of non-exposed cohort	Ascertainment of exposure	Outcome present at start of study	Comparability of cohorts	Assessment of outcome	Length of follow-up	Adequacy of follow-up	
Orazov [33]	NA	NA	*	*	*	*	*	*	6

## Discussion

We evaluated five single-arm studies that investigated various dosages of dydrogesterone and different treatment durations, and nine RCTs, four CCTs and one cohort study that compared the effect of various dosages of dydrogesterone with non-dydrogesterone therapies. This evidence mapping included 1709 female participants. All the participants had a diagnosis of endometriosis. Dydrogesterone was found to be more effective than gestrinone in relieving dysmenorrhea and achieving a higher pregnancy rate and was associated with a lower risk of adverse events such as elevated transaminase levels, vaginal dryness, and acne. Compared with GnRH-a, dydrogesterone was also associated with a lower risk of endometriosis recurrence and elevated transaminase levels. Whether there is a difference between dydrogesterone and leuprolide acetate, letrozole, and traditional Chinese medicine remains unclear due to insufficient data.

The above findings may be impacted by attrition bias and selective reporting in individual RCTs [35]. The randomization and blinding of outcome assessments were inadequately described in the original RCTs, which induced selection and detection bias. This bias also affected the quality of the meta-analyses. In addition, small sample sizes and unexplainable heterogeneity between studies also impacted the quality of the body of evidence, especially for outcomes such as pain relief, pregnancy rate, and adverse events. Certain included studies used the visual analogue score (VAS), while others used American Fertility Society (AFS) scores to measure improvement in endometriosis which is reflected in these outcomes. Owing to the shortcomings of the current VAS or AFS scores which are primarily descriptive classifications unrelated to biologic function, these measures may be inadequate to accurately assess improvement in endometriosis, especially long-term improvement.

There is very limited evidence for the effectiveness and safety of these drugs in the treatment of endometriosis due to the limited number of randomized controlled trials comparing each drug. However, a number of published clinical studies have provided evidence relevant to the pharmacological treatment of endometriosis. Synthetic progestogens have been shown to reduce AFS scores and provide pain relief, but the treatment does not improve fertility in women of reproductive age [37, 38]. There are only a few controlled clinical trials of dydrogesterone for the treatment of endometriosis which have shown symptomatic improvement with some evidence of objective improvement [16, 19]. Currently, guidelines for the management and diagnosis of endometriosis developed and funded by National Institute for Health and Care Excellence recommend the use of a combination oral contraceptive pill or a progestogen for women with suspected, confirmed, or recurrent endometriosis [10]. The European Society of Human Reproduction and Embryology (ESHRE) guidelines recommended progestagens or anti-progestagens (gestrinone) as one option to reduce endometriosis-associated pain (GRADE A). ESHRE guidelines also recommend the use of GnRH agonists (nafarelin, leuprolide, buserelin, goserelin or triptorelin) as an option for reducing endometriosis-associated pain, although evidence is limited regarding dosage and duration of treatment (GRADE A) [1].

Compared with gestrinone, GnRH agonists, and no treatment, dydrogesterone may be more effective in treating endometriosis. First, it does not suppress the normal endometrium or alter the natural progression of endometriosis, while causing atrophy of ectopic endometrium [20]. Second, most of the available evidence indicates that dydrogesterone does not inhibit ovulation and regular menstruation at the usual therapeutic dosages. Hence, patients are able to conceive while using dydrogesterone, if they so desire. Furthermore, dydrogesterone has not been shown to adversely affect embryos. Finally, dydrogesterone-associated side effects are rare as it has relatively low antagonistic activity at glucocorticoid and mineralocorticoid receptors compared with progesterone [14]. Consequently, weight gain and edema are not observed with dydrogesterone.

When investigating the effectiveness of progestogens and anti‐progestogens in the treatment of painful endometriosis, Brown et al. compared dydrogesterone with placebo and found no evidence of a difference in objective efficacy [36]. In contrast, Trivedi et al. found that pelvic pain, dysmenorrhea, and dyspareunia improved significantly after the first cycle of treatment with dydrogesterone in post-laparoscopic treatment of endometriosis [21].

At present, gestrinone is the only anti-progestagen that has been evaluated for the treatment of endometriosis. We did not identify any placebo-controlled trials or therapy trials comparing the efficacy of gestrinone. Only one review compared gestrinone with danazol [35] or a GnRH analogue (leuprorelin) [31] and found no evidence supporting a benefit of gestrinone over danazol. However, compared with gestrinone, a GnRH analogue (leuprorelin) significantly improved dysmenorrhea [31]. In this evidence mapping, dydrogesterone significantly improved pelvic pain and dysmenorrhea and lowered the occurrence of adverse events (elevated transaminase levels, vaginal dryness, and acne).

GnRH agonists, potentially useful for treating extensive endometriosis, function by rendering the patient hypoestrogenic thereby generating a condition of pseudomenopause. The pregnancy rate following treatment with GnRH agonists is not significantly different than that observed with ‘watchful waiting’ [39]. The major side effects of GnRH agonists are hot flushes, vaginal dryness, headaches, superficial dyspareunia, and a potential for the development of osteoporotic changes [15]. Furthermore, patients are not able to conceive while using GnRH agonists. Due to small sample sizes, we were unable to evaluate the differences between dydrogesterone and GnRH agonist treatment of endometriosis.

This study has a number of strengths. First, the search strategy was developed by professional information specialists who searched both electronic databases and the references of relevant systematic reviews, allowing the identification of a maximum number of relevant RCTs and CCTs. Second, the study screening and data extraction process were conducted by two researchers independently to minimize bias. Like all other studies, our evidence mapping also has some limitations. For instance, most of the included studies were published in journals with a lower impact factor, and there were some limitations in the design of these clinical studies. Specifically, the dose of dydrogesterone, the duration of therapy, and the criteria used to evaluate improvement of endometriosis were not consistent across the included studies. Consequently, primary and secondary outcomes’ data were insufficient to detect a clear difference between the groups. Wide confidence intervals were also noted for some results, and these data should be interpreted with caution [36]. Due to insufficient data, we failed to detect a difference between dydrogesterone and treatments with GnRH-a leuprolide acetate, letrozole, traditional Chinese medicine and acupuncture, or identical placebo.

Some further issues that were not discussed in this review should also be addressed in future studies. Although peritoneal superficial lesions and ovarian endometriomas represent the majority of endometriotic implants within the pelvis, deep infiltrating endometriosis and extra-pelvic endometriosis are the most challenging conditions to manage. Sometimes, medical therapy is sufficient to reduce symptoms [40]. However, a large number of patients may require an approach that entails complete eradication with a nerve and vascular sparing [41] to restore the normal pelvic anatomy and its functions.

## Conclusion

### Implication for practice

Dydrogesterone may be more effective in relieving pelvic pain and dysmenorrhea than gestrinone and appears to have fewer adverse effects. It may also be much safer to conceive while taking dydrogesterone. GnRH agonists have major side effects such as hot flushes, vaginal dryness, headaches, superficial dyspareunia, and a potential for the development of osteoporotic changes. Furthermore, conception should not be attempted while using this therapy. Compared to no treatment, dydrogesterone increases pregnancy rates during the first year after surgery, an increase with reaches statistical significance at 12 months.

### Implication for research

At present, there is limited high-quality research investigating commonly used treatments for endometriosis and comparing dydrogesterone with other hormonal treatments. In designing future trials, care should be taken to apply uniform standards for evaluating improvements in endometriosis and to ensure that all valuable and pertinent data are included, such as the results of surgical treatments (and other confounders) at the time of diagnosis and entry into the study.

## Conclusion

The amount and quality of evidence investigating the effects of dydrogesterone in the treatment of endometriosis is generally very low. Based on limited evidence, it is concluded that dydrogesterone may have some advantages over gestrinone, GnRH agonists, and other therapeutic interventions in the treatment endometriosis. However, this conclusion should be viewed with caution. The findings from this evidence mapping and meta-analysis could be of major importance for healthcare providers and researchers.

## Data Availability

Data are available from the authors upon reasonable request.

## Appendix 1

Search strategy in each database.

## Abbreviations

GnRH: Gonadotropin-releasing hormone
RCT: Randomized control trial
CCT: Clinical control trials
NOS: Newcastle–Ottawa Scale
VAS: Visual analogue score
AFS: American Fertility Society
